# Etymologia: *Legionella pneumophila*

**DOI:** 10.3201/eid2311.ET2311

**Published:** 2017-11

**Authors:** Ronnie Henry

**Keywords:** Legionella pneumophila, bacteria, pneumonia, convention, Joseph McDade, Charles C. Shepard

## *Legionella pneumophila* [leʺjə-nelʹə nooʺmo-filʹə]

In the summer of 1976, as the United States was celebrating the bicentennial of the Declaration of Independence, a mysterious acute respiratory illness developed in attendees at an American Legion convention in Philadelphia shortly after the attendees returned from the convention. In total, 182 Legionnaires became ill, and 29 died. 

Researchers in the Leprosy and Rickettsia Branch at Centers for Disease Control (CDC), headed by Charles C. Shepard, observed that guinea pigs became ill after being inoculated with lung tissues from patients who died. A few gram-negative bacilli were seen in guinea pig tissues, but these were believed to be normal flora or contaminants. The bacteria could not at first be isolated in embryonated eggs because the standard procedure for isolating rickettsiae at the time was to include penicillin and streptomycin to prevent contamination.

**Figure 1:**
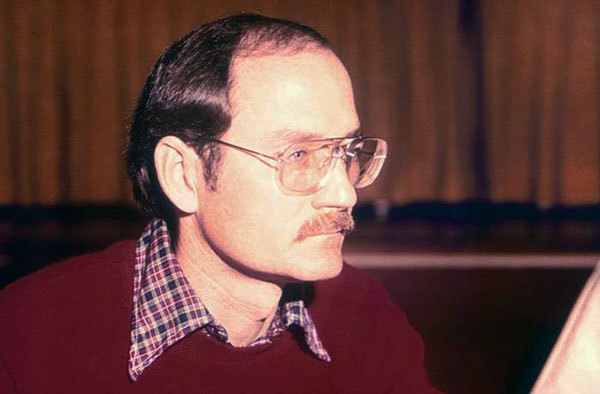
Joseph McDade, CDC scientist who discovered the cause of Legionnaires’ disease.

**Figure 2:**
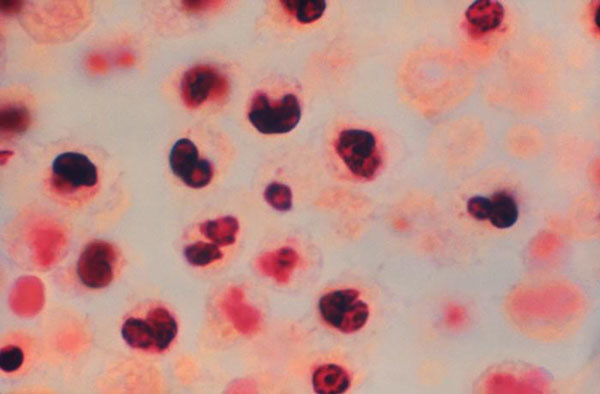
Lung cells with intra-alveolar exudate containing macrophages and polymorphonuclear leukocytes after infection with *Legionella pneumophila*, the causative agent of Legionnaires’ disease. Photos: McDade, R.E. Bates/CDC; photomicrograph, F.W. Chandler/CDC.

Returning to work after Christmas 1976, CDC microbiologist Joseph McDade (Figure 1) was bothered by these unexplained findings. He again attempted to grow the bacteria in embryonated eggs, this time without antibiotics, and successfully isolated a large inoculum of pure culture that could be grown on agar. These bacteria were determined to be the etiologic organism of Legionnaires’ disease and were eventually named *Legionella* (for the Legionnaires) *pneumophila* (Greek *pneumon* [lung] + *philos* [loving]) (Figure 2).
